# Type A personality, sleep quality, and cerebral small vessel disease: investigating the mediating role of sleep in a community-based study

**DOI:** 10.3389/fneur.2023.1236670

**Published:** 2023-08-03

**Authors:** Xirui Zhou, Hao Huang, Wensheng Qu, Zhiyuan Yu, Jing Zhao, Lingshan Wu, Yi Zhang, Qianqian Kong, Ziyue Wang, Xiang Luo

**Affiliations:** ^1^Department of Neurology, Tongji Hospital, Tongji Medical College, Huazhong University of Science and Technology, Wuhan, Hubei, China; ^2^Hubei Key Laboratory of Neural Injury and Functional Reconstruction, Huazhong University of Science and Technology, Wuhan, Hubei, China

**Keywords:** cerebral small vessel disease, type A behavior pattern, sleep quality, mediation effect, white matter hyperintensity

## Abstract

**Purpose:**

Type A behavior pattern (TABP) is a personality type characterized by rapid speech, impatience, competition, and hostility. Asymptomatic cerebral small vessel disease (CSVD) is often endemic in older adults. Individuals with TABP commonly experience suboptimal sleep quality, and a correlation exists between sleep disturbances and CSVD. We investigated the relationship between TABP and CSVD markers and further explored the mediating role of sleep quality in the relationship between TABP and CSVD.

**Methods:**

A cross-sectional survey included 764 community-dwelling adults aged 55–85 years. The TABP Scale and the Pittsburgh Sleep Quality Index (PSQI) were used to assess personality and sleep quality, respectively. Linear and logistic regression analyses were used to examine relationships between variables of interest. In addition, mediation analyses with bootstrapping were used to test whether sleep quality mediated the relationship between TABP and CSVD.

**Results:**

Of the 764 participants [median age 65 (61–69) years, 59.9% female], the population with type A personality accounted for 44.8%. After adjusting for covariates, TABP scores (*p* = 0.03) and PSQI scores (*p* < 0.001) were significantly correlated with CSVD. In addition, sleep quality partially mediated the association between type A behavior and CSVD, and the mediating effect was 10.67%.

**Conclusion:**

This study showed that type A behavior was a risk factor for CSVD among older community-dwelling adults and that sleep quality mediated the relationship between type A behavior and CSVD. Changing type A behavior may help improve sleep quality, which may in turn reduce the prevalence of CSVD.

## 1. Introduction

Cerebral small vessel disease (CSVD), common in the elderly, is a complex disease characterized by clinical, imaging, and pathological manifestations associated with small blood vessels in the brain (1). The imaging markers of CSVD include white matter hyperintensity (WMH), lacune (LA), enlarged perivascular space (EPVS), and cerebral microbleed (CMB) (2). Furthermore, CSVD may lead to adverse health events, including stroke, urinary disorders, dementia, and gait disturbances. Given that the population is aging, the burden of CSVD will increase rapidly. Therefore, it is urgent to clarify the pathogenesis of CSVD.

Type A behavior pattern (TABP) is an emotional complex characterized by time urgency, impatience, competition, and hostility and has high stability over the lifetime (3). Additionally, TABP may lead to sleep disturbances (4), mood disorders (5), and fall risk (6). Prior studies have also found that TABP is associated with cardiovascular disease (7) and multiple sclerosis (8). However, to the best of our knowledge, no study has examined whether TABP is associated with neuroimaging features of CSVD.

Physiological sleep is characterized by a cyclical progression of non-rapid eye movement (NREM) and rapid eye movement (REM) phases. NREM is characterized by a gradual reduction in neural sympathetic activity with parasympathetic predominance. Autonomic function during REM is comparable to that during wakefulness. Sleep disorders may have an impact on the autonomic nervous system, systemic hemodynamics, and endothelial function (9). Obstructive sleep apnea (OSA) is a prevalent primary sleep disorder, characterized by frequent episodes of upper airway obstruction during sleep and resulting in significant sleep fragmentation and deprivation (10). There is a potential causal association between OSA and hypertension, atherosclerosis, stroke, obesity, and metabolic syndrome (9).

Poor sleep is a common health problem in older adults. The quality of sleep affects both physiological and psychological processes in the body, and vice versa (11). A study demonstrated a significant correlation between physical activity, dietary habits, and sleep quality among athletes (12). Aerobic exercise has a beneficial impact on the sleep quality of individuals who are obese (13). In addition, there is a correlation between cognitive performance and sleep, both of which affect personality. One study showed that athletes may experience cognitive impairment as a result of partial sleep deprivation (14). Recently, several studies have demonstrated that insomnia, sleep apnea, and other sleep-related problems are positively related to CSVD characteristics, especially WMH (15, 16). In addition, prior research suggests that adults with TABP tend to have trouble falling asleep, experience more nightmares, and sleep less in comparison with those with type B behavior patterns (4). These studies suggest that sleep quality is associated with type A behavior and CSVD independently; however, the relationship between type A behavior, sleep quality, and CSVD is unclear.

In this study, we hypothesized that poor sleep quality would mediate the association between TABP and CSVD. A significant mediation effect would suggest that TABP contributes to the risk of CSVD due to sleep quality.

## 2. Materials and methods

### 2.1. Study population

The study population was selected from an ongoing community-based prospective cohort project aimed at investigating sporadic CSVD in the elderly population of Wuhan, China. The selection criteria for this project were based on previous cohort studies on CSVD (17–19). CSVD is a disease that exhibits age-related characteristics, and its incidence demonstrates an upward trend with increasing age. Therefore, the inclusion criteria were as follows (20): (1) aged 55–85 years; (2) able to complete the self-reported written questionnaire; (3) willing to participate in this study and provide informed consent. The cohort project aimed to investigate imaging biomarkers, cognitive function, gait performance, and emotional status in the elderly population with sporadic CSVD. Therefore, the exclusion criteria were as follows (21): (1) Parkinson's disease, Alzheimer's disease, or other neurodegenerative diseases; (2) severe mental disorders such as major depression and schizophrenia; (3) non-vascular diseases causing white matter lesions, carbon monoxide poisoning, multiple sclerosis, and adrenoleukodystrophy; (4) presence of cerebral hemorrhage, subarachnoid hemorrhage, intracranial space-occupying lesions, or acute ischemic cerebral infarction; (5) life expectancy < 3 years; (6) unable to complete the magnetic resonance imaging (MRI) examination due to MRI contraindications. We recruited the elderly population of the community through leaflet distribution, media publicity, and door-to-door outreach efforts. The head MRI and questionnaire assessment were completed within 1 day. A total of 764 community residents participated in this study. This study was approved by the Ethics Committee of Tongji Hospital, Tongji Medical College, Huazhong University of Science and Technology (No. 2019-S105).

### 2.2. TABP and sleep quality assessment

TABP was measured using the Chinese version of the TABP scale (22). The TABP scale, developed by the National Collaborative Group on Psychosomatic Medicine, consists of 60 items where participants are asked to answer “yes” or “no” to each item (5). The scale consists of three dimensions. First, the time hurry (TH) dimension reflects a sense of time urgency and speed of work. Second, the competition and hostility (CH) dimension represents competitiveness, hostility, and impatience. Third, the lie dimension score ≥7 indicates that the results of the scale are invalid (5). The overall score is significantly positively correlated with type A personality (5). A total score of ≥27 indicates TABP (22). Internal consistency reliability for the Chinese version of the TABP scale was 0.98, indicating good discriminant validity (22). Sleep quality was assessed using the Chinese version of the Pittsburgh Sleep Quality Index (PSQI) (23). The PSQI scale consists of 19 items, with a total score of 0–21. A PSQI score of >5 is considered poor sleep quality (24).

### 2.3. Covariate assessment

Demographic information and medical history were collected from all participants, including age, gender, body mass index (BMI), education level, smoking history, drinking history, stroke history, and whether they were diagnosed with hypertension, diabetes, hyperlipidemia, coronary heart disease, kidney disease, or any other disease (23). Symptoms of anxiety were measured using the Hamilton Anxiety Scale (HAMA). The Mini-Mental State Examination (MMSE) was used to assess cognitive functioning.

### 2.4. Magnetic resonance imaging

**Figure 1:**
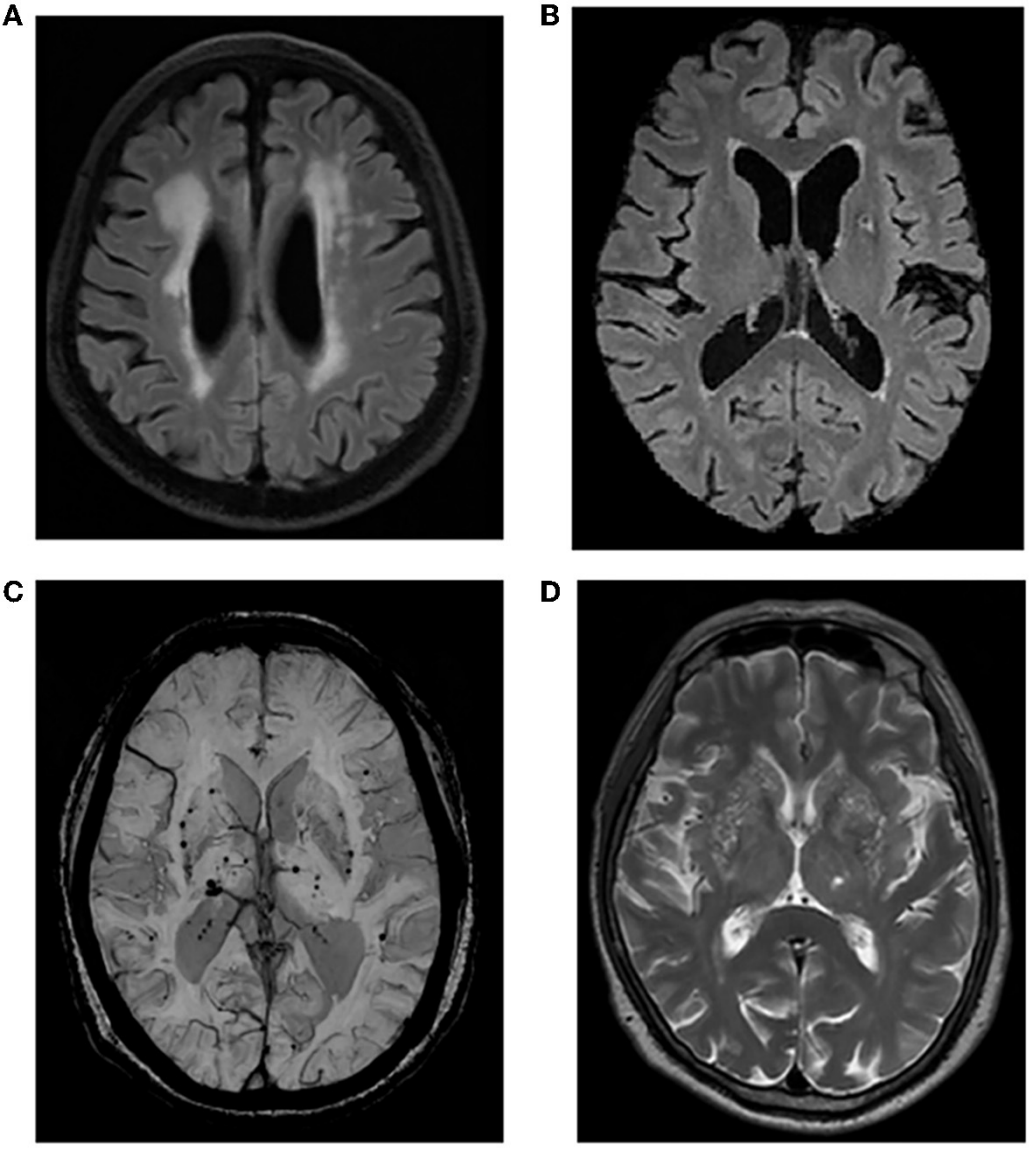
Representative imaging markers of CSVD. **(A)** Moderate-to-severe WMH; **(B)** LA in the left basal ganglia region; **(C)** CMBs in the basal ganglia; **(D)** moderate-to-severe EPVS in the basal ganglia. CSVD, cerebral small vessel disease; WMH, white matter hyperintensity; LA, lacune; CMB, cerebral microbleed; EPVS, enlarged perivascular space.

Brain MRI was obtained using a single 3T MRI scanner (United Imaging, Shanghai, China; see Supplementary Table 1 for detailed MRI protocols). Brain MRI included five sequences: T1-weighted, T2-weighted, fluid-attenuated inversion recovery (FLAIR), diffusion-weighted imaging (DWI), and susceptibility-weighted imaging (SWI). Two radiologists scored the neuroimaging markers for CSVD. Any disagreements were resolved by discussion with a superior physician. According to the Standards for Reporting Vascular Changes on Neuroimaging (STRIVE) (2): (1) WMH is characterized by irregular hyperintensity under the overlying cortex on the T2-weighted and FLAIR sequences. According to the Fazekas score, moderate-to-severe WMH included confluent lesions. (2) LA was defined as a subcortical ovoid with a cerebrospinal fluid-like signal ranging from 3 mm to 15 mm in diameter on all sequences. (3) CMB was characterized by a round low-signal void with a diameter of 2–10 mm on the SWI sequence. (4) EPVS was defined as an oval or linear cerebrospinal fluid-like signal < 3 mm in diameter on T2-weighted images. Grading was based on the number of gaps containing the largest EPVS in a unilateral basal segment (BG) section: 0 = no EPVS, 1 = 1–10 EPVS, 2 = 11–20 EPVS, 3 = 21–40 EPVS, 4 = ≥40 EPVS (25). A score of ≥2 was defined as moderate-to-severe EPVS. Total CSVD burden (0–4 points): moderate-to-severe WMH, any LA, any CMB, and moderate-to-severe EPVS (26) (Figure 1). A total burden of 0 indicates that CSVD is not present; otherwise, it is present (27).

### 2.5. Statistical analysis

Data were analyzed using SPSS version 23.0 software (SPSS Inc., Chicago, Illinois, USA). Variables with non-normal distribution were expressed as median values and quartiles (Q1–Q3), and count data were presented as percentages (%). The Mann–Whitney *U*-test or chi-square test was used to compare the differences between the two groups (TABP and non-TABP). Binary logistic regression was used to analyze the relationship between the TABP and PSQI scores with the presence of CSVD. Among them, the TABP or PSQI score was the independent variable, and the presence of CSVD (moderate-to-severe WMH, lacunar, CMB, and moderate-to-severe EPVS) was the dependent variable. Ordinal logistic regression was used to analyze the relationship between the TABP and PSQI scores and CSVD burden. Among them, the TABP or PSQI score was the independent variable, and the CSVD burden was the dependent variable. Linear regression was used to analyze the relationship between the TABP and PSQI scores. TABP was the independent variable, and the PSQI score was the dependent variable. Finally, the mediation analysis was performed using mediation packages in R, version 4.2.2 (R Foundation for Statistical Computing, Vienna, Austria) (28). Among them, TABP was the independent variable, the PSQI score was the mediator variable, and the presence of CSVD (moderate-to-severe WMH, lacunar, CMB, and moderate-to-severe EPVS) was the dependent variable. To obtain robust effect estimates, the number of bootstrap samples was set to 5,000. Age, sex, education, HAMA score, and vascular risk factors (hypertension, diabetes, hyperlipidemia, and BMI) were entered as covariates. A *p*-value of < 0.05 was defined as statistically significant.

## 3. Results

### 3.1. Demographic and clinical data

**Figure 2:**
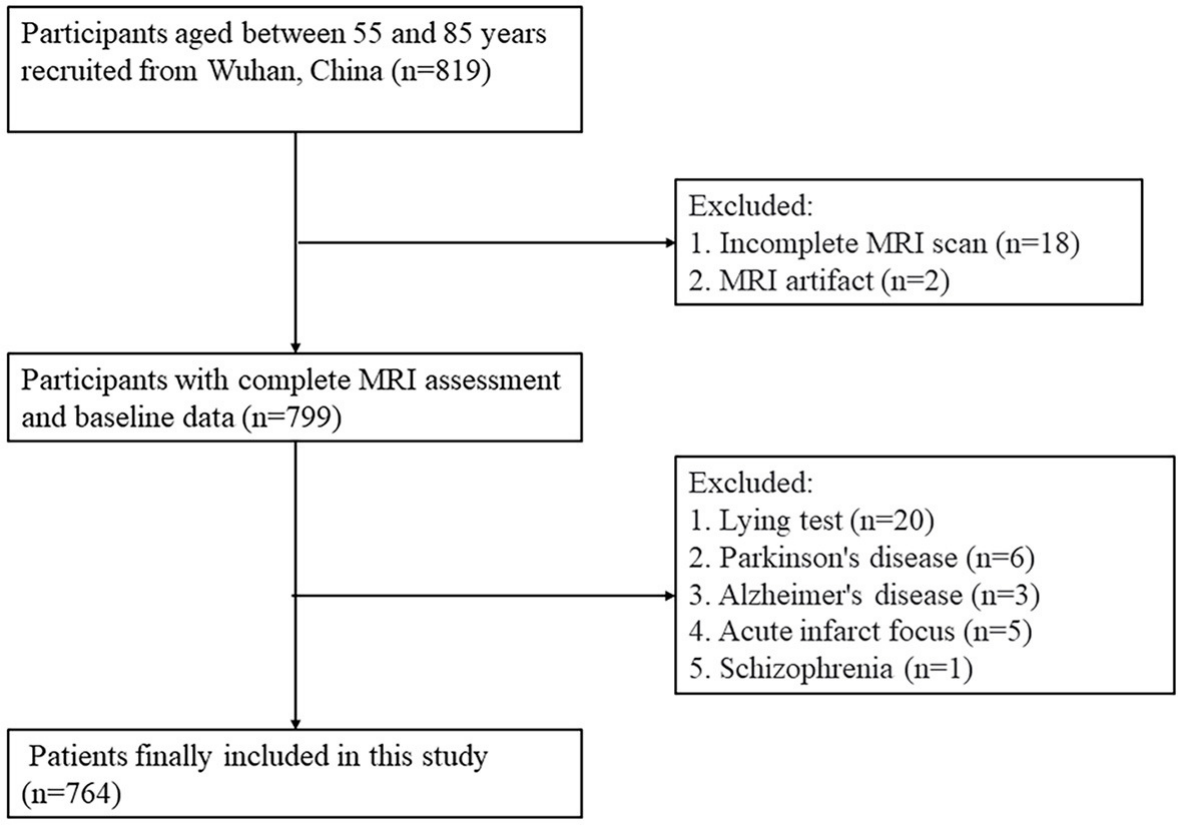
Flowchart of the study.

A total of 764 subjects were included in this study (Figure 2). The overall median age was 65 (range: 61–69) years, 458 cases (59.9%) were female, and 342 cases (44.76%) were TABP. The TABP group had a lower education level, higher BMI, a higher proportion of CSVD and diabetes, higher HAMA scores, lower MMSE scores, and poorer sleep quality (Table 1). There were no significant differences in age, hypertension, hyperlipidemia, smoking, and drinking history between the two groups (Table 1).

**Table 1:** Comparison of clinical variables in TABP patients.

	**Total (764)**	**No-TABP (422)**	**TABP (342)**	***p*-value**
Age, years, median (IQR)	65 (61–69)	65 (61–69)	66 (61–70)	0.494
Female, *n* (%)	458 (59.9%)	267 (63.3%)	191 (55.8%)	**0.037**
Education level, median (IQR)	12 (11–16)	12 (12–16)	12 (9–15)	<**0.001**
TABP score, median (IQR)	26 (20–32)	20 (17–24)	30 (30–37)	<**0.001**
TH score, median (IQR)	13 (10–16)	10 (8–12)	17 (15–19)	<**0.001**
CH score, median (IQR)	13 (10–16)	10 (8–12)	16 (15–18)	<**0.001**
MMSE, median (IQR)	28 (27–29)	29 (28–29)	28 (27–29)	<**0.001**
PSQI score, median (IQR)	7 (4–10)	6 (4–9)	7 (5–11)	<**0.001**
**Vascular risk factors**				
BMI, median (IQR)	24 (22–26)	23 (21–25)	24 (22–26)	**0.006**
Smoking history, *n* (%)	85 (11.1%)	41 (9.7%)	44 (12.9%)	0.169
Drinking history, *n* (%)	83 (10.9%)	42 (10.0%)	41 (12.0%)	0.369
Diabetes, *n* (%)	119 (15.6%)	49 (11.6%)	70 (20.5%)	**0.001**
Hypertension, *n* (%)	343 (44.9%)	178 (42.2%)	165 (48.2%)	0.094
Hyperlipidemia, *n* (%)	238 (31.2%)	126 (29.9%)	112 (32.7%)	0.391
HAMA, median (IQR)	3 (1–5)	3 (1–4)	2 (3–5)	<**0.001**
**Neuroimaging characteristics**				
CSVD, *n* (%)	317 (41.5%)	144 (34.1%)	173 (50.6%)	<**0.001**
Moderate-to-severe WMH, *n* (%)	144 (18.8%)	52 (12.3%)	92 (26.9%)	<**0.001**
Presence of LA, *n* (%)	88 (11.5%)	40 (9.5%)	48 (14.0%)	0.050
Presence of CMB, *n* (%)	150 (19.6%)	71 (16.8%)	79 (23.1%)	**0.030**
>10 EPVS-BG, *n* (%)	134 (17.5%)	61 (14.5%)	73 (21.3%)	**0.013**

TABP, type A behavior pattern; SD, standard deviation; TH, time hurry; CH, competition and hostility; MMSE, Mini-Mental state examination; PSQI, Pittsburgh sleep quality index; BMI, body mass index; HAMA, Hamilton anxiety scale; HAMD, Hamilton depression scale; CSVD, cerebral small vessel disease; WMH, white matter hyperintensity; LA, lacune; CMB, cerebral microbleed; EPVS-BG, enlarged perivascular space—basal ganglia.

Bold values mean *p*-value of < 0.05.

### 3.2. Associations between key variables

After adjustment for age, sex, education, HAMA score, and vascular risk factors, an ordinal regression analysis showed that the TABP score and the PSQI score were positively associated with CSVD burden, indicating that participants with higher TABP scores or poorer sleep quality had a greater CSVD burden (Table 2). Linear regression analyses showed that the higher the TABP score, the worse the sleep quality (Table 3). In addition, binary logistic regression analyses showed that TABP and sleep quality were associated with the presence of CSVD (Table 4), moderate-to-severe WMH (Supplementary Table 2), and moderate-to-severe EPVS (Supplementary Table 2). However, TABP was not associated with the presence of LA and CMB (Supplementary Table 2).

**Table 2:** Ordinal logistic regression analyses with CSVD burden as the DV.

	**Unadjusted model**			**Adjusted model**		
	**OR**	**95% CI**	***p*** **-value**	**OR**	**95% CI**	***p*** **-value**
TABP score	1.039	1.020–1.057	**< 0.001**	1.030	1.011–1.049	**0.002**
TH score	1.053	1.020–1.087	**0.001**	1.040	1.005–1.075	**0.024**
CH score	1.074	1.040–1.108	**< 0.001**	1.058	1.022–1.094	**0.001**
PSQI score	1.080	1.040–1.121	**< 0.001**	1.116	1.066–1.168	**< 0.001**

Adjusted for age, gender, education level, HAMA scale, BMI, hypertension, diabetes, and hyperlipidemia.

CSVD, cerebral small vessel disease; DV, dependent variable; CI, confidence interval; TABP, type A behavior pattern; TH, time hurry; CH, competition and hostility; PSQI, Pittsburgh sleep quality index.

Bold values mean *p*-value < 0.05.

**Table 3:** Multivariate linear regression models with the PSQI score as the DV.

	**Unadjusted model**			**Adjusted model**		
	**OR**	**95% CI**	***p*** **-value**	**OR**	**95% CI**	***p*** **-value**
TABP score	1.092	1.058–1.127	**< 0.001**	1.040	1.010–1.070	**0.009**
TH score	1.164	1.100–1.234	**< 0.001**	1.064	1.010–1.120	**0.020**
CH score	1.143	1.079–1.213	**< 0.001**	1.065	1.011–1.122	**0.018**

Adjusted for age, gender, education level, HAMA scale, BMI, hypertension, diabetes, and hyperlipidemia.

PSQI, Pittsburgh sleep quality index; DV, dependent variable; CI, confidence interval; TABP, type A behavior pattern; TH, time hurry; CH, competition and hostility.

Bold values mean *p*-value < 0.05.

**Table 4:** Binary logistic regression analyses with the presence of CSVD as the DV.

	**Unadjusted model**			**Adjusted model**		
	**OR**	**95% CI**	***p*** **-value**	**OR**	**95% CI**	***p*** **-value**
TABP score	1.036	1.018–1.056	**< 0.001**	1.031	1.010–1.052	**0.003**
TH score	1.048	1.014–1.083	**0.005**	1.037	1.001–1.075	**0.044**
CH score	1.073	1.037–1.109	**< 0.001**	1.063	1.025–1.102	**0.001**
PSQI score	1.069	1.027–1.112	**0.001**	1.097	1.044–1.153	**< 0.001**

Adjusted for age, gender, education level, HAMA scale, BMI, hypertension, diabetes, and hyperlipidemia.

CSVD, cerebral small vessel disease; DV, dependent variable; CI, confidence interval; TABP, type A behavior pattern; TH, time hurry; CH, competition and hostility; PSQI, Pittsburgh sleep quality index.

Bold values mean *p*-value < 0.05.

### 3.3. Mediating effect of sleep quality on TABP and CSVD

**Figure 3:**
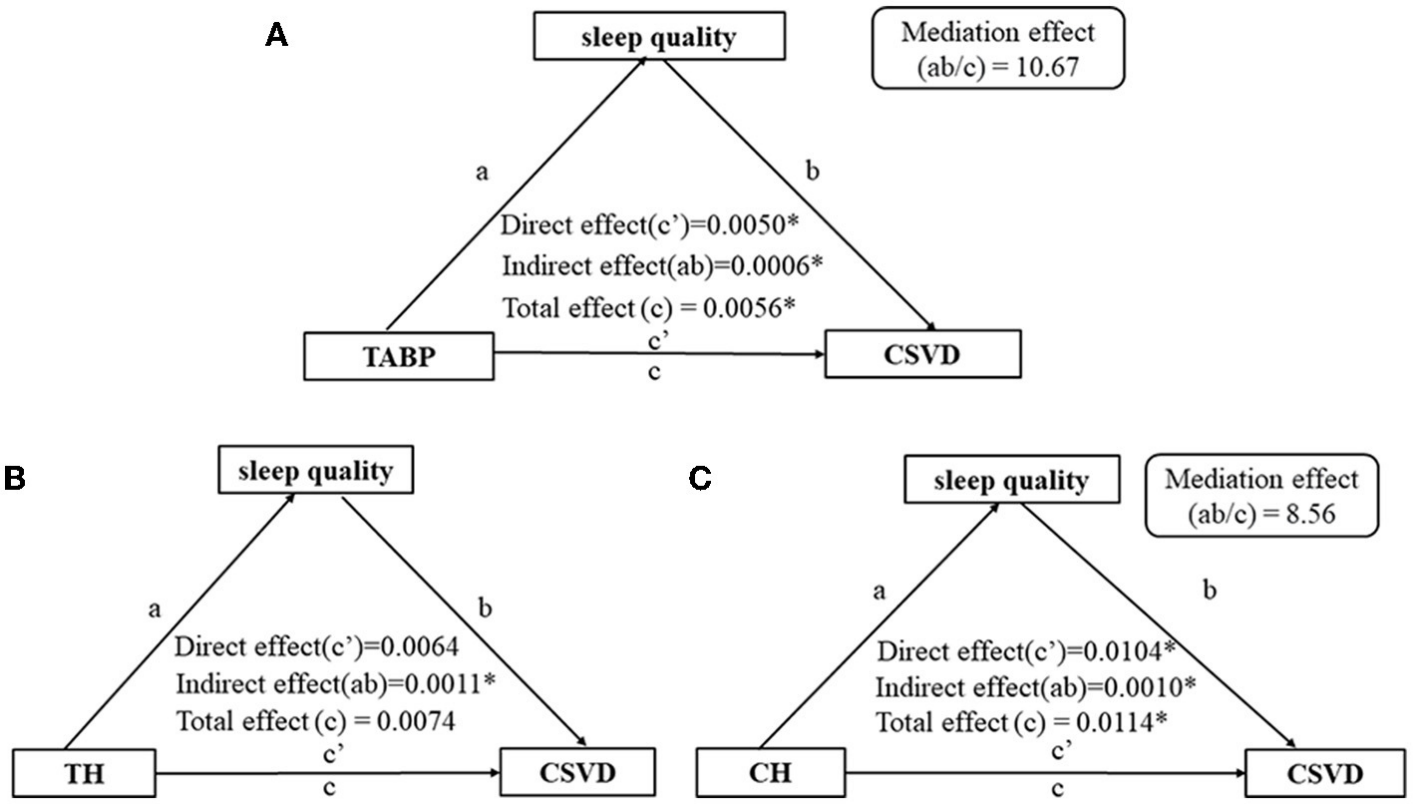
Path diagram of the relationship between type A behavior and the presence of CSVD, with sleep quality as the mediator. **(A)** Path diagram of the relationship between TABP and CSVD, with sleep quality as the mediator. **(B)** Path diagram of the relationship between TH and CSVD, with sleep quality as the mediator. **(C)** Path diagram of the relationship between CH and CSVD, with sleep quality as the mediator. *p-value of < 0.05. Control variables: age, gender, education level, HAMA scale, BMI, hypertension, diabetes, and hyperlipidemia. CSVD, cerebral small vessel disease; TABP, type A behavior pattern; TH, time hurry; CH, competition and hostility.

**Figure 4:**
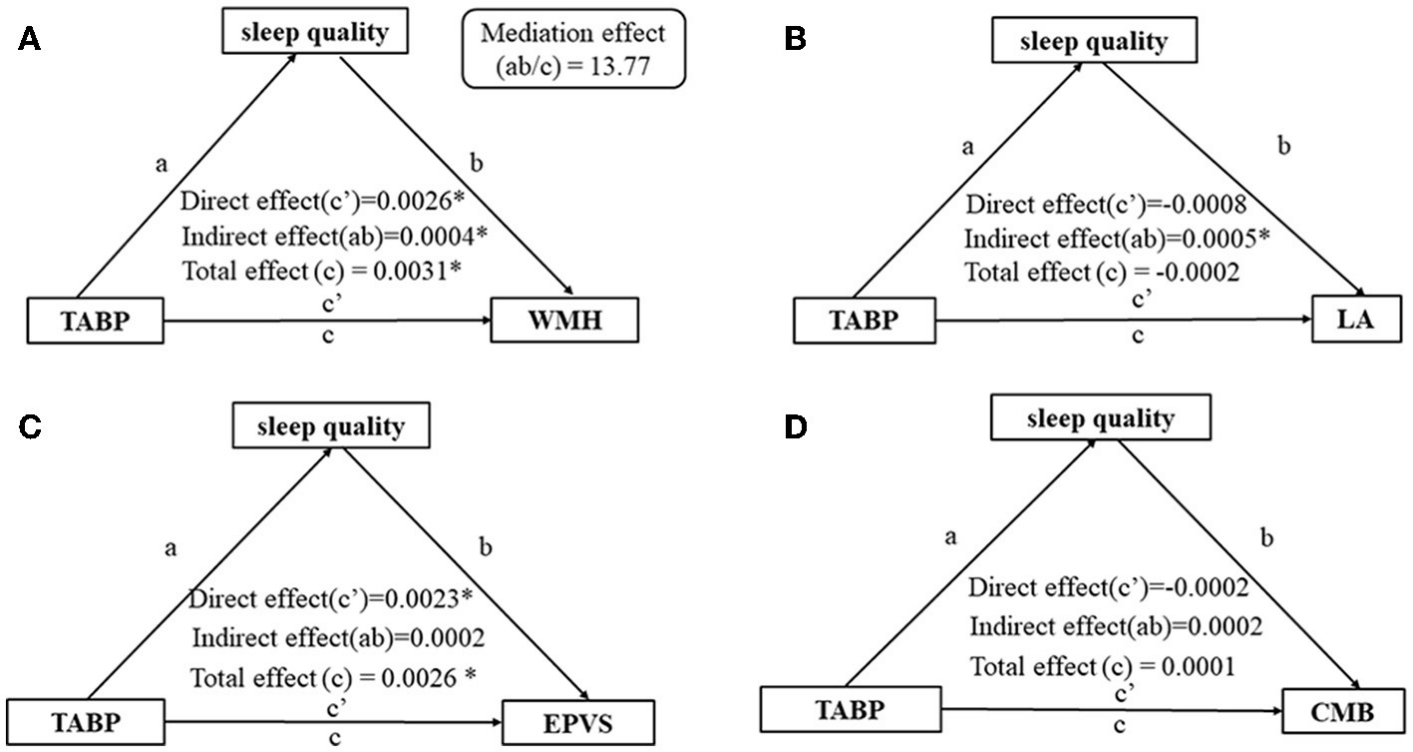
Analysis of the mediating effect of sleep quality on the relationship between TABP and the presence of CSVD markers. **(A)** Analysis of the mediating effect of sleep quality on the relationship between TABP and moderate-to-severe WMH. **(B)** Analysis of the mediating effect of sleep quality on the relationship between TABP and the presence of LA. **(C)** Analysis of the mediating effect of sleep quality on the relationship between TABP and moderate-to-severe EPVS. **(D)** Analysis of the mediating effect of sleep quality on the relationship between TABP and the presence of CMB. ^*^p-value of < 0.05. Control variables: age, gender, education level, HAMA scale, BMI, hypertension, diabetes, and hyperlipidemia. TABP, type A behavior pattern; CSVD, cerebral small vessel disease; WMH, white matter hyperintensity; LA, lacune; CMB, cerebral microbleed; EPVS, enlarged perivascular space.

The total effect (*c* = 0.0056, *p* < 0.01), direct effect (*c*′ = 0.0050, *p* = 0.01), and indirect effect (path *a* × path *b* = 0.0006, *p* = 0.02) of TABP on the presence of CSVD were statistically significant, after adjusting for age, gender, education level, HAMA score, and vascular risk factors (Figure 3A). The mediating effect *ab*/*c* was 10.67% (Figure 3A). Furthermore, sleep quality mediated the relationship between the CH dimension and CSVD (Figure 3C) but not the relationship between TH and CSVD (Figure 3B). Additionally, sleep quality mediated the relationship between TABP and WMH (Figure 4A) but did not mediate the relationship between TABP and other CSVD imaging markers (Figure 4).

## 4. Discussion

The results from the present study demonstrated a positive association between TABP and CSVD in community-dwelling older adults, and sleep quality mediated this association. The findings of this study suggest that older adults with TABP, especially those with higher CH scores, may be at risk for sleep disorders, which may further influence CSVD. Furthermore, worse sleep quality partially mediated the association between TABP and moderate-to-severe WMH.

CSVD burden has been widely used to assess the severity of CSVD (26, 29). In the present study, the CSVD group (scores 1–4) accounted for 41.5% of the total population. This prevalence was slightly higher than the results of a previous study (33.8%) that included 1,586 community adults over the age of 35 in China (29). A possible reason for the different findings is that the present study enrolled older adults. The TABP scale is a widely used scale to assess type A behavior (5, 30). Individuals with high TABP scores have been found to be quick and diligent but often irritable, impatient, and aggressive (30). Higher TABP scores were significantly associated with more severe depressive disorders, higher frustration, and higher work stress (31, 32).

Although TABP was associated with coronary heart disease and multiple sclerosis (8, 33), no study has examined the relationship between TABP and CSVD burden. The present study found that TABP was significantly associated with CSVD burden. Many studies have found that TABP was associated with hypertension (34), hyperlipidemia (35), diabetes (36), alcohol consumption (37), and atherosclerosis (35), all of which may increase the risk of CSVD burden. We also found that TABP was positively correlated with WMH and EPVS but not LA and CMB, which may be due to the different pathogenesis of the four CSVD markers. The current literature describes WMH as a demyelinating lesion with chronic hypoperfusion of blood flow due to atherosclerosis of the vessel wall and restriction of the lumen (38). The mechanism of EPVS remains unclear. Furthermore, EPVS has been described as an indicator of inadequate drainage of the glymphatic system, elevated cerebral venous pressure, and neuroinflammation (39). EPVS and WMH may overlap in pathogenesis (39). Additionally, LA has been proposed to originate from an ischemic or hemorrhagic stroke but lacked definitive symptoms and may be associated with cerebral amyloid lesions (39). Finally, CMB has been described as a perivascular hemosiderin deposition, indicating that inflammation and endothelial dysfunction are important parts of the pathogenesis (39). Type A personality is associated with chronic stress, which has adverse effects on health behavior, contributes to cerebrovascular atherosclerosis (35), and promotes the development of WMH and EPVS.

Consistent with previous studies, our results suggested that sleep quality was associated with TABP (5, 32, 40) and CSVD (16, 41–43). Several previous studies have suggested that sleep quality may lead to CSVD through decreased cerebral perfusion, increased cerebrovascular resistance, impaired vasomotor responsiveness, sympathetic tone, insulin resistance, inflammatory activation, and increased blood–brain barrier permeability (43, 44). Some studies have shown that adults with higher TABP scores have worse sleep quality (4, 5, 32, 40). TABP may contribute to sleep disorders due to impaired self-regulatory stress systems, abnormal dopamine expression, and other factors (4, 5, 32, 40). Another study demonstrated that high TABP scores were significantly associated with depressive symptoms, high frustration levels, and high levels of work-related stress (3). High TABP may contribute to small vessel ischemia and hypoxia by stimulating the neuroendocrine system and activating the sympathetic nervous system, resulting in active catecholamine secretion, increased vascular resistance, and elevated blood pressure level (45). The results from our study suggest that sleep quality mediated the relationship between competition and hostility (CH) and CSVD but not time urgency (TH) and CSVD, possibly due to different pathogenesis.

This study highlights the critical role of TABP in the development of sleep quality and CSVD in community-dwelling older adults. There is a need for TABP assessment in older adults in the community because TABP can be treated. A twin study of type A behavior suggests that TABP comprises 45% genetic and 55% environmental factors, which would be valuable for prevention and treatment (46). First, cognitive behavioral therapy and health education may help TABP populations identify adverse and maladaptive behaviors and replace them with more adaptive behaviors (34). Second, those with TABP may relieve stress and bad moods through meditation or music therapy. Alternatively, β-blockers alter adrenergic responsiveness, which is considered an important physiological trait of type A personality (47). Finally, trazodone may treat sleep disorders, thereby alleviating the cognitive impairment caused by CSVD (42).

There were several limitations in the present study. First, this study was cross-sectional and therefore cannot establish a causal association between TABP and CSVD. Second, the TABP self-rated scale was completed by participants; therefore, the data may include recall bias. Future studies with stronger research designs should explore the relationship between personality traits and CSVD. Third, the primary limitation of this study is the lack of use of precise sleep measurement tools such as polysomnography. Sleep quality was measured using a self-rated scale and thus may be affected by self-report. Fourth, the generalization to the overall population should be approached with caution due to the lack of random sampling in this project. Finally, future researchers should conduct longitudinal studies on the relationship between TABP, sleep quality, and CSVD.

## 5. Conclusion

This study demonstrated that markers of CSVD, moderate-to-severe WMH and EPVS, were more prevalent in individuals with higher TABP scores among community-dwelling older adults. In addition, sleep quality mediated the association between TABP and CSVD. We did not investigate the effectiveness of behavioral interventions on sleep quality or CSVD prevention, therefore, further research is needed in this area.

## Data availability statement

The original contributions presented in the study are included in the article/Supplementary material, further inquiries can be directed to the corresponding author.

## Ethics statement

The studies involving human participants were reviewed and approved by the Ethics Committee of Tongji Hospital (No. 2019-S105). The patients/participants provided their written informed consent to participate in this study.

## Author contributions

XZ drafted and revised the manuscript. HH and WQ studied concept and design. ZY revised the manuscript. JZ and LW processed the statistical data. YZ, QK, and ZW collected the clinical data. XL designed and guided the study. All authors contributed to the article and approved the submitted version.
