# Supplementary material for: Type A personality, sleep quality, and cerebral small vessel disease: investigating the mediating role of sleep in a community-based study
Source: Front Neurol. 2023 Aug 3;14:1236670. doi: 10.3389/fneur.2023.1236670 (PMC10437815; doi:10.3389/fneur.2023.1236670)
Supplement: Supplementary file 1 [file Data_Sheet_1.pdf]

*Supplementary Material*

# Type A Personality, Sleep Quality, and Cerebral Small Vessel Disease: Investigating the Mediating Role of Sleep in a Community-Based Study

**Supplemental Table 1: Parameters of MRI sequences**

| MRI sequence | Voxel size (mm) | FOV (mm) | TR (ms) | TE (ms) | Flip angle (deg) | Slice thickness (mm) |
|---|---|---|---|---|---|---|
| T1W | 1×1×1 | 240×220 | 5.9 | 2.5 | 8 | 1 |
| T2W | 1×1×1 | 240×220 | 3400 | 314.16 | NA | 1 |
| FLAIR | 1×1×1 | 240×220 | 8000 | 518 | NA | 1 |
| SWI | 0.72×0.72×2 | 230×230 | 30 | 20 | 15 | 2 |
| DWI | 1.44×1.44×3 | 230×230 | 10113 | 73.6 | 90 | 3 |

Notes: MRI = magnetic resonance imaging; FOV = field of view; TR = repetition time; TE = echo time; T1W = T1-weighted; T2W = T2-weighted; FLAIR = fluid-attenuated inversion recovery; SWI = susceptibility-weighted imaging; DWI = diffusion-weighted image; NA = not available.

**Supplementary Table 2. Binary logistic regression analyses with the presence of CSVD markers as the DV.**

| | Unadjusted model | | | Adjusted model | | |
|---|---|---|---|---|---|---|
| | OR | 95% CI | P | OR | 95% CI | P |
| **moderate-to-severe WMH** | | | | | | |
| TABP score | 1.055 | 1.030–1.079 | **< 0.001** | 1.049 | 1.023–1.076 | **0.001** |
| TH score | 1.098 | 1.053–1.145 | **< 0.001** | 1.096 | 1.047–1.148 | **< 0.001** |
| CH score | 1.082 | 1.038–1.128 | **< 0.001** | 1.065 | 1.019–1.114 | **0.006** |
| PSQI score | 1.117 | 1.065–1.172 | **< 0.001** | 1.199 | 1.127–1.275 | **< 0.001** |
| **moderate-to-severe EPVS** | | | | | | |
| TABP score | 1.043 | 1.018–1.068 | **0.001** | 1.033 | 1.008–1.060 | **0.011** |
| TH score | 1.058 | 1.015–1.104 | **0.009** | 1.044 | 0.997–1.093 | 0.065 |
| CH score | 1.083 | 1.037–1.131 | **< 0.001** | 1.065 | 1.017–1.114 | **0.007** |
| PSQI score | 1.074 | 1.022–1.128 | **0.004** | 1.080 | 1.018–1.147 | **0.011** |
| **LA** | | | | | | |
| TABP score | 1.016 | 0.988–1.044 | 0.273 | 0.997 | 0.967–1.027 | 0.832 |
| TH score | 1.017 | 0.967–1.068 | 0.516 | 0.988 | 0.935–1.043 | 0.660 |
| CH score | 1.035 | 0.984–1.088 | 0.182 | 1.001 | 0.950–1.056 | 0.959 |
| PSQI score | 1.115 | 1.053–1.181 | **< 0.001** | 1.154 | 1.075–1.238 | **< 0.001** |
| **CMB** | | | | | | |
| TABP score | 1.004 | 0.983–1.027 | 0.694 | 1.001 | 0.978–1.025 | 0.932 |
| TH score | 1.000 | 0.961–1.040 | 0.995 | 0.998 | 0.956–1.041 | 0.910 |
| CH score | 1.015 | 0.975–1.056 | 0.471 | 1.006 | 0.964–1.050 | 0.790 |
| PSQI score | 1.027 | 0.979–1.077 | 0.271 | 1.042 | 0.984–1.103 | 0.159 |

Adjusted for age, gender, education level, HAMA scale, BMI, hypertension, diabetes, and hyperlipidemia.

CSVD = Cerebral small vessel disease; DV = dependent variable; WMH = White matter hyperintensity; EPVS = Enlarged perivascular space; LA = Lacune; CMB = cerebral microbleed; CI = Confidence interval; TABP = Type A behavior pattern; TH = Time hurry; CH = Competition and hostility; PSQI = Pittsburgh sleep quality index.

Bold values mean $p < 0.05$.
